# Prognostic Capability of TNBC 3-Gene Score among Triple-Negative Breast Cancer Subtypes

**DOI:** 10.3390/cancers14174286

**Published:** 2022-09-01

**Authors:** Jhajaira M. Araujo, Gabriel De la Cruz-Ku, Melanie Cornejo, Franco Doimi, Richard Dyer, Henry L. Gomez, Joseph A. Pinto

**Affiliations:** 1 Centro de Investigación Básica y Traslacional, AUNA Ideas, Lima 15036, Peru; 2 Escuela Profesional de Medicina Humana, Universidad Privada San Juan Bautista, Lima 15067, Peru; 3 Department of Surgery, University of Massachusetts, Worcester, MA 01604, USA; 4 Universidad Cientifica del Sur, Lima 15067, Peru; 5 Departamento de Patología, Oncosalud-AUNA, Lima 15036, Peru; 6 Departamento de Medicina Oncológica, Oncosalud-AUNA, Lima 15036, Peru

**Keywords:** TNBC, prognostic biomarkers, survival, DRFS

## Abstract

**Simple Summary:**

In this study we evaluated the prognostic capability of the 3-gene score in the molecular subtypes of triple negative breast cancer and found that the score was able to predict the risk of distant recurrence in the immunomodulatory and mesenchymal stem-like subtypes. Additionally, a low 3-gene score was related to a high level of tumor-infiltrating lymphocytes. Our findings suggest that the prognostic capability of the 3-gene score is associated to tumor-infiltrating components.

**Abstract:**

*Background:* Triple-negative breast cancer (TNBC) is a complex and molecularly heterogeneous entity, with the poorest outcome compared with other breast cancer subtypes. Previously, we developed a TNBC 3-gene score with a significant prognostic capability. This study aims to test the 3-gene score in the different TNBC subtypes. *Methods:* Data from 204 TNBC patients treated with neoadjuvant chemotherapy were retrieved from public datasets and pooled (GSE25066, GSE58812, and GSE16446). After removing batch effects, cases were classified into Lehman’s TNBC subtypes and then the TNBC 3-gene score was used to evaluate the risk of distant recurrence in each subgroup. In addition, the association with tumor-infiltrating lymphocyte (TILs) levels was evaluated in a retrospective group of 72 TNBC cases. *Results:* The TNBC 3-gene score was able to discriminate patients with different risks within the pooled cohort (HR = 2.41 for high vs. low risk; 95%CI: 1.50–3.86). The score showed predictive capability in the immunomodulatory subtype (HR = 4.16; 95%CI: 1.63–10.60) and in the mesenchymal stem-like subtype (HR = 18.76; 95%CI: 1.68–208.97). In the basal-like 1, basal-like-2, and mesenchymal subtypes, the observed differential risk patterns showed no statistical significance. The score had poor predictive capability in the luminal androgen receptor subtype (*p* = 0.765). In addition, a low TNBC 3-gene score was related to a high level of TIL infiltration (*p* < 0.001). *Conclusions:* The TNBC 3-gene score is able to predict the risk of distant recurrence in TNBC patients, specifically in the immunomodulatory and mesenchymal stem-like subtype. Despite a small sample size in each subgroup, an improved prognostic capability was seen in TNBC subtypes with tumor-infiltrating components.

## 1. Introduction

Triple-negative breast cancer (TNBC) is a term coined to define a group of breast cancers lacking the expression of an estrogen receptor (ER), a progesterone receptor (PR), and a human epidermal growth factor receptor 2 (HER2) [1]. From a pathological viewpoint, TNBC cases are characterized for being more aggressive than other subtypes due to their high histological grade and presence of compromised lymph nodes at the time of diagnosis [2]. Furthermore, TNBC represents 10–20% of all breast cancers, with a higher prevalence in young and pre-menopausal patients than in older patients [1,3,4]. In addition, African American and Hispanic patients show a higher prevalence of TNBC in contrast to Caucasian and Asian women [5].

At present, neoadjuvant chemotherapy (NAC) is the most effective treatment and standard of care for non-metastatic TNBC, with high rates of clinical and pathologic response and an improved outcome among responders [6,7]. Nevertheless, not all patients show the same responses or survival rates, which suggests that TNBC is molecularly heterogeneous [2,8,9].

In 2011, Lehmann et al., classified the TNBC into six molecular subtypes. These subtypes include the basal-like 1 (BL1), characterized by its rapid cell division, high proliferation rate seen as Ki67 greater than 70%, lack of cell cycle control, and high DNA damage response, especially in the ATR/BRCA gene pathways; however, this subtype showed to have the best prognosis. The basal-like 2 (BL2), with altered growth factor signaling, activation of glycolysis and gluconeogenesis routes, and high expression of growth factor receptors; the immunomodulatory (IM), with a strong molecular signature of immune cell processes such as high T cell, B cell, chemokine and NF-kappa B signaling pathways; the mesenchymal (M) and mesenchymal stem-like (MSL), while both present a high expression of genes involved in cell motility, cellular differentiation and growth pathways, the MSL expresses a different group of growth (platelet-derived growth factor, epidermal growth factor receptor, G-protein coupled receptor signaling) and angiogenic factors (vascular endothelial growth factor 2, tyrosine kinase with immunoglobulin-like EGF-like domains 1), low levels of proliferation genes as well as claudins, and high levels of stem cell factors; the M subtype encodes pathways involved in the cell cycle, mismatch repair, DNA damage, osteocyte and adipocyte genes; the luminal androgen receptor (LAR), characterized by the expression of androgen receptors, low proliferation, elevated steroid hormone synthesis, and high androgen and estrogen metabolism, despite being ER negative, this subtype is also the most chemo resistant but has a favorable prognosis [10,11,12,13,14,15].

Previously, we developed a linear predictor for distant recurrence-free survival (DRFS) based on the expression of three genes (*CCL5*, *DDIT4*, and *POLR1C*) [16] by conducting an analysis of the expression levels of 449 genes related to TNBC aggressiveness. In addition, we reported that a high *DDIT4* expression was related to a poor outcome in different types of cancer, the dysregulation of POLR1C gene expression is involved in tumor aggressiveness in breast cancer, while *CCL5*, typically associated with a poor outcome, in TNBC is associated with a major concentration of tumor-infiltrating lymphocytes (TILs) and recruitment of CD8 T-cells, CD4 activated T-cells, NK activated cells, and M1 macrophages [17,18]. Furthermore, it has been demonstrated that high levels of TILs are associated with better disease-free survival, overall survival, and response to chemotherapy as well as immunotherapy [19,20].

Due to the molecular heterogeneity of TNBC, our aim was to evaluate the prognostic capability of the TNBC 3-gene scores in the six molecular subtypes of TNBC, which may be useful in the development of a tailored therapeutic approach for TNBC. As a secondary objective, we analyzed the relation between the prognostic signature and TIL infiltration.

## 2. Methods

### 2.1. Patients

We included TNBC patients treated with neoadjuvant chemotherapy (NAC) to evaluate the prognostic capability of the TNBC 3-gene score. Patients were selected from three public datasets available at Gene expression Omnibus (GEO) (https://www.ncbi.nlm.nih.gov/geo/ accessed on 15 June 2021) [21].

**GSE25066:** A total of 113 TNBC cases (determined by immunohistochemistry) with residual disease (RD) after NAC were selected. Gene expression profiling was measured with the U133A Affymetrix microarray platform (Affymetrix, Santa Clara, CA, USA) (https://www.ncbi.nlm.nih.gov/geo/query/acc.cgi?acc=gse25066 accessed on 15 June 2021) [22].

**GSE58812:** A total of 107 TNBC samples with unknown response to the neoadjuvant treatment were evaluated. Gene expression was profiled with the Affymetrix Human Genome U133 Plus 2.0 Array (Affymetrix, Santa Clara, CA, USA) (https://www.ncbi.nlm.nih.gov/geo/query/acc.cgi?acc=GSE58812 accessed on 15 June 2021) [23].

**GSE16446:** A total of 47 ER- and HER2-negative samples with RD were identified. Gene expression was profiled with the Affymetrix Human Genome U133 Plus 2.0 Array (Affymetrix, Santa Clara, CA, USA) (https://www.ncbi.nlm.nih.gov/geo/query/acc.cgi?acc=gse16446 accessed on 15 June 2021) [24].

In addition, we included a retrospective cohort of 74 TNBC Peruvian patients with residual disease after NAC, with TIL count information. Since this dataset was profiled with Nanostring and TNBC subtype information was not available, it was not included in the metabase. Gene expression profile and TIL assessment of this cohort have been previously described [18].

### 2.2. Subtype Identification

The online tool TNBCtype (https://cbc.mc.vanderbilt.edu/tnbc/ accessed on 15 June 2021) [25] was used to classify the samples of the public datasets according to the TNBC subtypes. In datasets with more than one probe for the same gene, values were collapsed to the highest level of gene expression.

Samples identified as possible ER positive were removed and the analysis was repeated. In total, 22 samples were excluded from GSE25066, 11 samples from GSE58812, and one sample from GSE16446.

### 2.3. Elaboration of the Metabase

Since the unstable subtype (UNS) was not considered a Lehmann’s TNBC molecular subtype, these patients were removed and then the datasets were pooled. Eleven samples were removed from GSE25066, thirteen samples from GSE58812, and five samples from GSE16446.

The remaining samples in the three datasets, GSE25066 (*n* = 80), GSE58812 (*n* = 83), GSE16446 (*n* = 41), were combined into one and transformed to base 2 logarithm (log2) and centered by the median. The online tool COMBAT V3, implemented in Genepattern, was used to eliminate the batch effect in the metabase [26].

To verify that there was no batch effect in the metabase, we used the F-test of the analysis of variance (ANOVA) and a graphical method based on linear discriminant analysis (LDA).

### 2.4. Prognostic Capability of the TNBC 3-Gene Score According to TNBC Subtypes

The TNBC 3-gene score was calculated according to the following formula: −0.393 × CCL5 + 0.443 × DDIT4 + 0.490 × POLR1C, as reported in Pinto et al., (2016) [16]. The median was used as the cutoff to establish groups with a high risk (values higher than the median) or a low risk (values equal or lower than the median) of recurrence.

Distant recurrence-free survival (DRFS) was estimated with the Kaplan–Meier method and Log-rank or Breslow tests were used to compare survival curves. Hazard ratios were estimated by the Cox proportional hazards model, evaluating the risk score as a categorical variable.

The risk score was compared between TNBC subtypes using the ANOVA test and Tukey’s multiple comparison test.

### 2.5. Evaluation of the Relation between TNBC 3-Gene Score and TILs

The TNBC 3-gene score was evaluated as continuous variable while TIL count was categorized into high and low, using a cutoff value of 20% since it has been proved as a prognostic biomarker of survival in TNBC [18,27,28]. The boxplot graphic and the Student’s t-test were used to analyze the differences between groups.

## 3. Results

### 3.1. TNBC Subtypes

**Figure 1:**
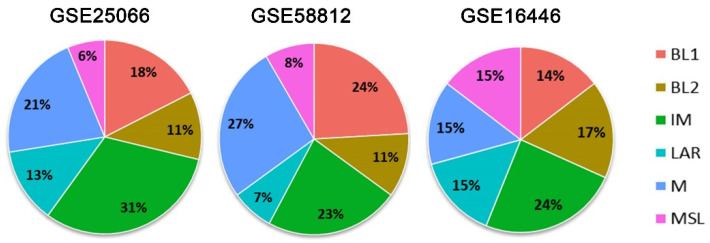
Distribution of Triple Negative Breast Cancer subtypes in each dataset, GSE25066 (*n* = 80), GSE58812 (*n* = 83) and GSE16446 (*n* = 41). Abbreviations: basal-like 1 (BL1), basal-like 2 (BL2), immunomodulatory (IM), mesenchymal (M), mesenchymal stem-like (MSL), and luminal androgen receptor (LAR).

In total, 204 patients were included. The distribution of patients according to the TNBC subtypes was: BL1, 19.6% (*n* = 40); BL2, 12.3% (*n* = 25); IM, 26.5% (*n*= 54); LAR, 10.8% (*n* = 22); M, 22.1% (*n* = 45); MSL, 8.8% (*n* = 18). The frequency of TNBC subtypes in each dataset is shown in Figure 1.

### 3.2. Gene Expression of CCL5, DDIT4, and POLR1C in the Metabase

After using COMBAT v3 to eliminate the batch effect in the metabase, the expression of genes *CCL5* (*p* = 0.869), *DDIT4* (*p* = 0.830), and *POLR1C* (*p* = 0.991) did not present significant differences between the three databases. Furthermore, the linear discriminant function (LDA) plot did not show grouping in the data (Figure S1).

### 3.3. Predictive Value of the TNBC 3-Gene Score in the Metabase

**Figure 2:**
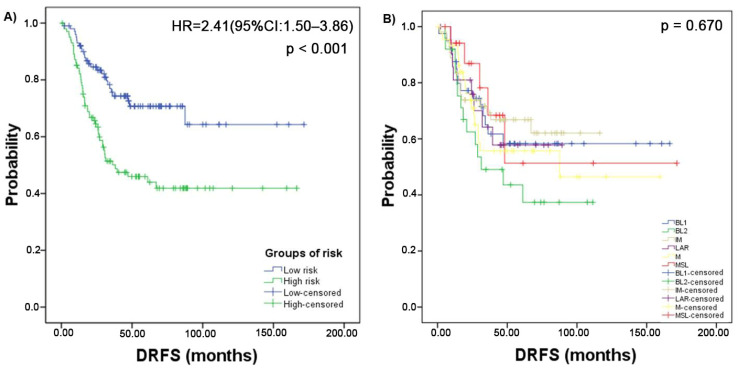
Survival curves of 204 Triple negative breast cancer (TNBC) patients. (**A**) Distant recurrence free survival (DRFS) of TNBC patients stratified by the 3-genes score using the median as cutoff. (**B**) DRFS for patients stratified by molecular TNBC subtype. Abbreviations: basal-like 1 (BL1), basal-like 2 (BL2), immunomodulatory (IM), mesenchymal (M), mesenchymal stem-like (MSL), and luminal androgen receptor (LAR).

The median value of the score risk (0.9863) was used as a cutoff to establish two groups with different risk of recurrence. A statistically significant difference was observed, with a 5-year DRFS of 70.7% for the low-risk group and 46.0% for the high-risk group (*p* < 0.001). The CoxPH analysis showed a HR = 2.41 (95%CI:1.50–3.86; *p* < 0.001) for recurrence in the high-risk group (Figure 2).

### 3.4. Three-Gene Score Predictive Value in TNBC Subtypes and Relation with TILs

**Figure 3:**
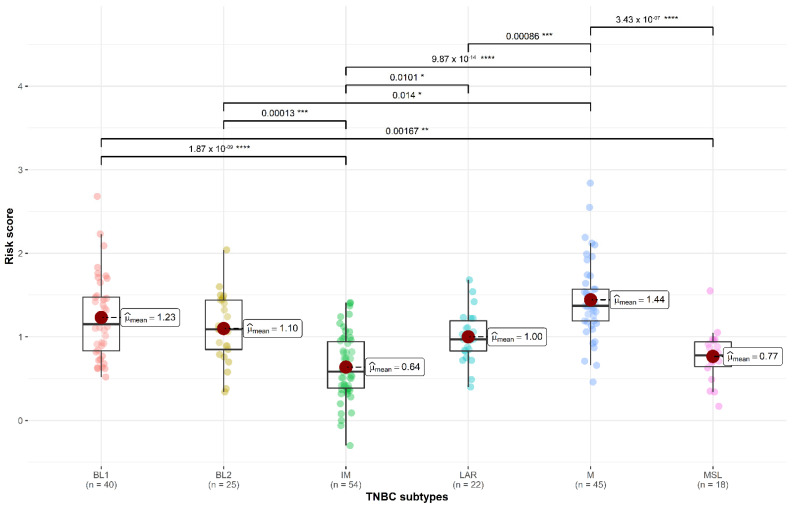
Distribution of the 3-genes score in each TNBC subtype. Lower values were observed in the IM and MSL subtype. Asterisks represent statistical significance (* *p* < 0.05, ** *p* < 0.01, *** *p* < 0.001, **** *p* < 0.0001).

The risk score presented a significant difference in relation to the molecular subtype of the TNBC (*p* < 0.001) and was lower in patients with IM and MSL subtypes. The risk score of IM was significantly lower than BL1 (*p* < 0.001), LAR (*p* = 0.010), and M (*p* < 0.001), while the risk score of MSL was lower than BL1 (*p* = 0.002) and M (*p* < 0.001) (Figure 3).

**Figure 4:**
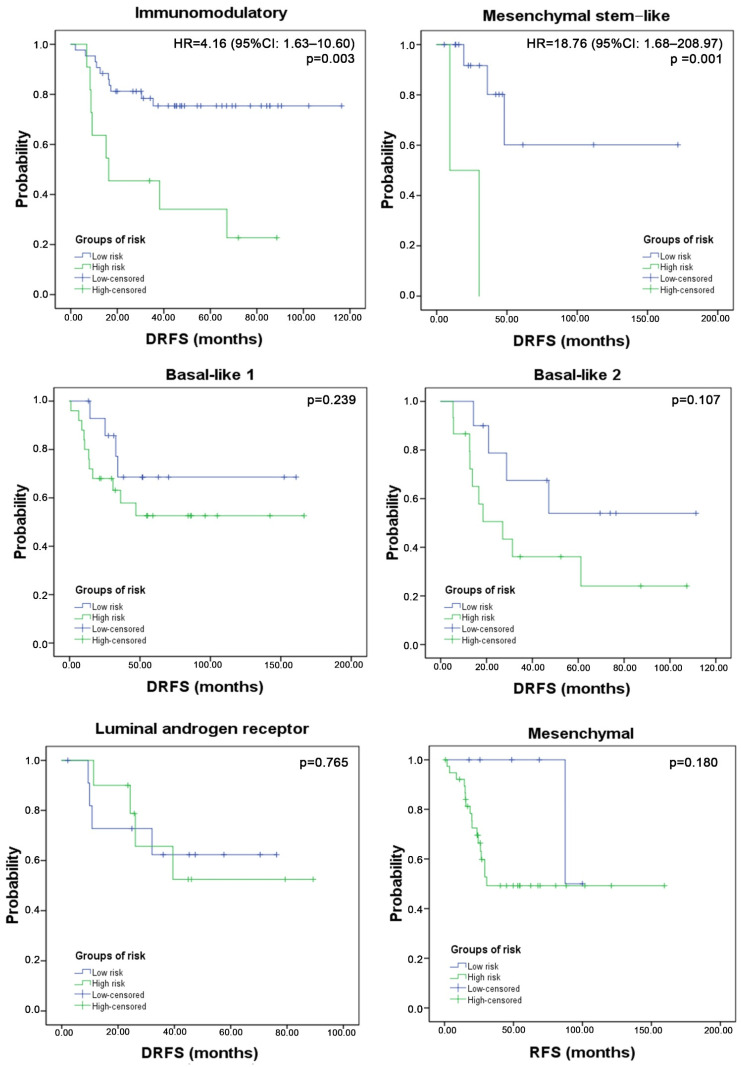
Three genes signature as prognostic factor of DRFS in TNBC subtypes. The 3 genes-score was statistically significant in the immunomodulatory and mesenchymal stem-like subtypes.

The TNBC 3-gene score was able to discriminate groups with different risk of recurrence only in the IM and MSL subtypes. The hazard ratios were 4.16 (95%CI: 1.63–10.60; *p* = 0.003) and 18.76 (95%CI: 1.68–208.97; *p* = 0.017) for the IM and MSL, respectively. Survival curves and *p* values are shown in Figure 4.

**Figure 5:**
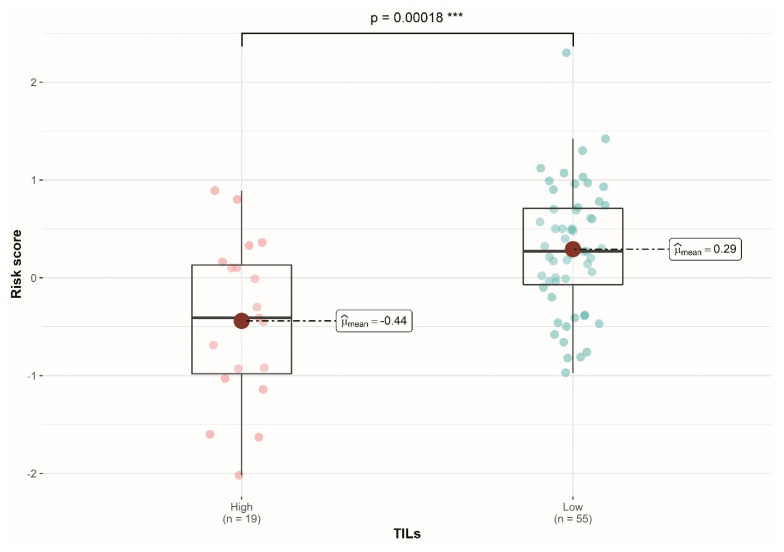
Comparison of the Three genes risk score according to TILs group. Patients with low TILs had a higher score. Asterisks represent statistical significance level (*** *p* < 0.001).

A high-risk score was shown in patients with low TILs. Differences between groups were statistically significant (*p* < 0.001) (Figure 5).

## 4. Discussion

In this study, the TNBC 3-gene prognostic signature was able to predict the risk of recurrence in the different subtypes of TNBC, specifically in the immunomodulatory and mesenchymal stem-like subtype. Moreover, among patients without complete pathologic response to NAC, a lower risk score was associated with high levels of tumor infiltrating components.

TNBC is a group of molecularly heterogeneous breast tumors that can be grouped into six different subtypes (BL1, BL2, IM, M, MSL, and LAR) by the expression level of approximately 1500 genes [10]. To date, there are limited options to use targeted therapy, despite advances in the understanding of this disease. TNBC patients with resistance to neoadjuvant chemotherapy have worse prognosis than patients achieving complete response, who show similar outcomes to non-TNBC patients [15]. In contrast, TNBC patients exhibit an increased risk of recurrence up to three years after the surgery, after which the risk of recurrence decreases dramatically [9]. These features describe the unmet need for biomarkers to stratify patients according to their risk and for new molecular targets to develop better therapeutic strategies.

Our previous research reported a TNBC 3-gene signature, based on the expression of *DDIT4*, *POLR1C*, and *CCL5*, which was able to discriminate TNBC patients at different risks of recurrence. This linear score was developed in tumors resistant to neo-adjuvant chemotherapy and was tested in three independent datasets of TNBC cases, where untreated tumors were assessed with microarrays [16].

In this work, we pooled data of three independent TNBC datasets and determined the TNBC subtypes, with the goal of testing our TNBC 3-gene signature on each subtype. Despite the fact that Lehman et al., (2016) [29] corrected their subtype classifications because of stromal and immune cell contamination leading to false classification of the MSL and IM subtypes, respectively, we decided to include them as subgroups in the evaluation of the TNBC 3-gene signature, due to the possible impact of the percentage of lymphocytes infiltration within the tumor on the prognosis of these patients, therefore leading to significant changes in the 3-gene signature results [14,30].

We observed clear differences between risk groups in the IM and MSL subtypes, while BL1, BL2, and M subtypes presented statistical trends. Interestingly, the lowest 3-gene score was seen in IM and MSL subtypes and these subtypes presented high infiltration of immune and stromal cells. Therefore, we concluded that the tumor microenvironment might have a strong influence on the predictive capability of the TNBC 3-gene score, which could be the explanation for the low-risk score among these subtypes [31]. In fact, during the last decades, several genomic predictors have been developed for TNBC and estrogen-negative tumors, and they share inclusion of immune or microenvironment-related gene sets [32,33,34]. For instance, Loi et al., reported that in early node-negative TNBC, TILs of ≥30% improved invasive DFS compared with <30% at 5-year follow-up (88% vs. 81%). This finding was corroborated in a meta-analysis which showed that high levels of TILs had better short term and long-term prognoses, specifically for CD4^+^, CD8^+^, and FOXP3^+^ [20,35].

We demonstrated that the 3-gene TNBC score is related to TIL infiltration, where a lower score was associated with a high infiltration of TILs. This observation might be explained by the influence of *CCL5* [36,37]. The expression of this gene causes a lowering of the score and, biologically, participates in the recruitment of TILs in TNBC [16,18].

On the other hand, the TNBC 3-gene signature had a poor discriminative performance in LAR cases (*p* = 0.765). The LAR tumors have a high expression of the androgen receptor and levels of androgen receptor-mediated signaling. LAR tumors are biologically characterized by a low Ki-67 index and better outcomes than androgen receptor- negative tumors [38]. Our prognostic signature was based on TNBC with residual disease, therefore it was made in tumors with some grade of resistance to chemotherapy and high replication rates, which could be an explanation of the lack of risk differentiation in the LAR subtype due to its biology and overall better prognosis compared with other TNBC subtypes [16,39].

Teschendorff et al., (2007) found that the prognosis in estrogen-positive genes are associated with an expression of cell cycle genes, while, in estrogen-negative cases, prognosis is related to the expression of genes involved in the immune response pathways [40]. Rody et al., (2011), in an unsupervised clustering of data from 579 TNBC cases, found that signatures related to a high B-cell infiltration and low IL8 levels were related to better prognoses in terms of event free survival [41]. Criscitiello et al., (2018) developed a signature based on the expression of four genes to predict lymphocyte infiltration and, consequently, the ability to predict patients at different risks of death and distant recurrence. In addition, expressions of immune genes were also related with the response to neoadjuvant chemotherapy [42].

The main limitation of our study was the small sample size in each TNBC subtype (ranging from 18 to 54 cases), leading to an unpowered analysis. Despite this, clear patterns were shown in the RFS analysis. Moreover, the retrospective design of our study can lead to bias in the interpretation of our results, although we controlled several variables with the purpose to have a uniform cohort; for this reason, new randomized controlled trials assessing the TNBC 3-gene prognostic signature are needed. Furthermore, although our pooled cohort includes all TNBC patients who underwent NAC independently from the pathologic response to chemotherapy, further studies are needed that include patients divided by complete pathological response, partial response, and no response.

## 5. Conclusions

In conclusion, the TNBC 3-gene score had an improved performance of predicting the risk of recurrence in IM and MSL TNBC subtypes. This score, based only on the expression of three genes, could be useful in clinical practice to stratify TNBC patients according to their risk, particularly in cases with TILs. Further randomized controlled trials are needed to validate this score in TNBC patients and their subtypes.

## Supplementary Materials

The following are available online at https://www.mdpi.com/article/10.3390/cancers14174286/s1, Figure S1: Distribution of CCL5, DDIT4 and POLR1C gene expression in three datasets, GSE25064 (*n* = 80), GSE58812 (*n*=83) and GSE16446 (*n* = 41).

## References

[1] Bauer K.R., Brown M., Cress R.D., Parise C.A., Caggiano V. (2007). Descriptive Analysis of Estrogen Receptor (ER)-Negative, Progesterone Receptor (PR)-Negative, and HER2-Negative Invasive Breast Cancer, the so-Called Triple-Negative Phenotype. Cancer.

[2] Haffty B.G., Yang Q., Reiss M., Kearney T., Higgins S.A., Weidhaas J., Harris L., Hait W., Toppmeyer D. (2006). Locoregional Relapse and Distant Metastasis in Conservatively Managed Triple Negative Early-Stage Breast Cancer. J. Clin. Oncol..

[3] Vallejos C.S., Gómez H.L., Cruz W.R., Pinto J.A., Dyer R.R., Velarde R., Suazo J.F., Neciosup S.P., León M., de la Cruz M.A. (2010). Breast Cancer Classification According to Immunohistochemistry Markers: Subtypes and Association With Clinicopathologic Variables in a Peruvian Hospital Database. Clin. Breast Cancer.

[4] Carey L.A., Perou C.M., Livasy C.A., Dressler L.G., Cowan D., Conway K., Karaca G., Troester M.A., Chiu K.T., Edmiston S. (2006). Race, Breast Cancer Subtypes, and Survival in the Carolina Breast Cancer Study. JAMA.

[5] Morris G.J., Naidu S., Topham A.K., Guiles F., Xu Y., McCue P., Schwartz G.F., Park P.K., Rosenberg A.L., Brill K. (2007). Differences in Breast Carcinoma Characteristics in Newly Diagnosed African–American and Caucasian Patients. Cancer.

[6] Yao L., Pang Z., Wang M., Wang M., Sun X., Cui M., Zheng Y., Li X., Dong H., Zhang Q. (2022). The Choice of a Neoadjuvant Chemotherapy Cycle for Breast Cancer Has Significance in Clinical Practice: Results from a Population-Based, Real World Study. Cancer Biol. Med..

[7] Cortazar P., Zhang L., Untch M., Mehta K., Costantino J.P., Wolmark N., Bonnefoi H., Cameron D., Gianni L., Valagussa P. (2014). Pathological Complete Response and Long-Term Clinical Benefit in Breast Cancer: The CTNeoBC Pooled Analysis. Lancet.

[8] Dent R., Trudeau M., Pritchard K.I., Hanna W.M., Kahn H.K., Sawka C.A., Lickley L.A., Rawlinson E., Sun P., Narod S.A. (2007). Triple-Negative Breast Cancer: Clinical Features and Patterns of Recurrence. Clin. Cancer Res..

[9] Liedtke C., Mazouni C., Hess K.R., André F., Tordai A., Mejia J.A., Symmans W.F., Gonzalez-Angulo A.M., Hennessy B., Green M. (2008). Response to Neoadjuvant Therapy and Long-Term Survival in Patients With Triple-Negative Breast Cancer. J. Clin. Oncol..

[10] Lehmann B.D., Bauer J.A., Chen X., Sanders M.E., Chakravarthy A.B., Shyr Y., Pietenpol J.A. (2011). Identification of Human Triple-Negative Breast Cancer Subtypes and Preclinical Models for Selection of Targeted Therapies. J. Clin. Investig..

[11] Burstein M.D., Tsimelzon A., Poage G.M., Covington K.R., Contreras A., Fuqua S.A.W., Savage M.I., Osborne C.K., Hilsenbeck S.G., Chang J.C. (2015). Comprehensive Genomic Analysis Identifies Novel Subtypes and Targets of Triple-Negative Breast Cancer. Clin. Cancer Res..

[12] Hubalek M., Czech T., Müller H. (2017). Biological Subtypes of Triple-Negative Breast Cancer. Breast Care.

[13] Ensenyat-Mendez M., Llinàs-Arias P., Orozco J.I.J., Íñiguez-Muñoz S., Salomon M.P., Sesé B., DiNome M.L., Marzese D.M. (2021). Current Triple-Negative Breast Cancer Subtypes: Dissecting the Most Aggressive Form of Breast Cancer. Front. Oncol..

[14] Yin L., Duan J.J., Bian X.W., Yu S.C. (2020). Triple-Negative Breast Cancer Molecular Subtyping and Treatment Progress. Breast Cancer Res..

[15] Santonja A., Sánchez-Muñoz A., Lluch A., Chica-Parrado M.R., Albanell J., Chacón J.I., Antolín S., Jerez J.M., de la Haba J., de Luque V. (2018). Triple Negative Breast Cancer Subtypes and Pathologic Complete Response Rate to Neoadjuvant Chemotherapy. Oncotarget.

[16] Pinto J.A., Araujo J., Cardenas N.K., Morante Z., Doimi F., Vidaurre T., Balko J.M., Gomez H.L. (2016). A Prognostic Signature Based on Three-Genes Expression in Triple-Negative Breast Tumours with Residual Disease. NPJ Genomic Med..

[17] Pinto J.A., Rolfo C., Raez L.E., Prado A., Araujo J.M., Bravo L., Fajardo W., Morante Z.D., Aguilar A., Neciosup S.P. (2017). In Silico Evaluation of DNA Damage Inducible Transcript 4 Gene (DDIT4) as Prognostic Biomarker in Several Malignancies. Sci. Rep..

[18] Araujo J.M., Gomez A.C., Aguilar A., Salgado R., Balko J.M., Bravo L., Doimi F., Bretel D., Morante Z., Flores C. (2018). Effect of CCL5 Expression in the Recruitment of Immune Cells in Triple Negative Breast Cancer. Sci. Rep..

[19] El Bairi K., Haynes H.R., Blackley E., Fineberg S., Shear J., Turner S., de Freitas J.R., Sur D., Amendola L.C., Gharib M. (2021). The Tale of TILs in Breast Cancer: A Report from The International Immuno-Oncology Biomarker Working Group. NPJ Breast Cancer.

[20] Gao G., Wang Z., Qu X., Zhang Z. (2020). Prognostic Value of Tumor-Infiltrating Lymphocytes in Patients with Triple-Negative Breast Cancer: A Systematic Review and Meta-Analysis. BMC Cancer.

[21] Barrett T., Wilhite S.E., Ledoux P., Evangelista C., Kim I.F., Tomashevsky M., Marshall K.A., Phillippy K.H., Sherman P.M., Holko M. (2012). NCBI GEO: Archive for Functional Genomics Data Sets—Update. Nucleic Acids Res..

[22] Hatzis C., Pusztai L., Valero V., Booser D.J., Esserman L., Lluch A., Vidaurre T., Holmes F., Souchon E., Wang H. (2011). A Genomic Predictor of Response and Survival Following Taxane-Anthracycline Chemotherapy for Invasive Breast Cancer. JAMA.

[23] Jézéquel P., Loussouarn D., Guérin-Charbonnel C., Campion L., Vanier A., Gouraud W., Lasla H., Guette C., Valo I., Verrièle V. (2015). Gene-Expression Molecular Subtyping of Triple-Negative Breast Cancer Tumours: Importance of Immune Response. Breast Cancer Res..

[24] Desmedt C., Di Leo A., de Azambuja E., Larsimont D., Haibe-Kains B., Selleslags J., Delaloge S., Duhem C., Kains J.-P., Carly B. (2011). Multifactorial Approach to Predicting Resistance to Anthracyclines. J. Clin. Oncol..

[25] Chen X., Li J., Gray W.H., Lehmann B.D., Bauer J.A., Shyr Y., Pietenpol J.A. (2012). TNBCtype: A Subtyping Tool for Triple-Negative Breast Cancer. Cancer Inform..

[26] Johnson W.E., Li C., Rabinovic A. (2007). Adjusting Batch Effects in Microarray Expression Data Using Empirical Bayes Methods. Biostatistics.

[27] Sun P., He J., Chao X., Chen K., Xu Y., Huang Q., Yun J., Li M., Luo R., Kuang J. (2021). A Computational Tumor-Infiltrating Lymphocyte Assessment Method Comparable with Visual Reporting Guidelines for Triple-Negative Breast Cancer. EBioMedicine.

[28] Pruneri G., Vingiani A., Bagnardi V., Rotmensz N., De Rose A., Palazzo A., Colleoni A.M., Goldhirsch A., Viale G. (2016). Clinical Validity of Tumor-Infiltrating Lymphocytes Analysis in Patients with Triple-Negative Breast Cancer. Ann. Oncol. Off. J. Eur. Soc. Med. Oncol..

[29] Lehmann B.D., Jovanović B., Chen X., Estrada M.V., Johnson K.N., Shyr Y., Moses H.L., Sanders M.E., Pietenpol J.A. (2016). Refinement of Triple-Negative Breast Cancer Molecular Subtypes: Implications for Neoadjuvant Chemotherapy Selection. PLoS ONE.

[30] Espinosa Fernandez J.R., Eckhardt B.L., Lee J., Lim B., Pearson T., Seitz R.S., Hout D.R., Schweitzer B.L., Nielsen T.J., Rayne Lawrence O. (2020). Identification of Triple-Negative Breast Cancer Cell Lines Classified under the Same Molecular Subtype Using Different Molecular Characterization Techniques: Implications for Translational Research. PLoS ONE.

[31] Zheng H., Siddharth S., Parida S., Wu X., Sharma D. (2021). Tumor Microenvironment: Key Players in Triple Negative Breast Cancer Immunomodulation. Cancers.

[32] Guo M., Wang S.M. (2021). Genome Instability-Derived Genes Are Novel Prognostic Biomarkers for Triple-Negative Breast Cancer. Front. Cell Dev. Biol..

[33] Qin Y., Deng J., Zhang L., Yuan J., Yang H., Li Q. (2021). Tumor Microenvironment Characterization in Triple-Negative Breast Cancer Identifies Prognostic Gene Signature. Aging (Albany NY).

[34] Wang X., Su W., Tang D., Jing J., Xiong J., Deng Y., Liu H., Ma W., Liu Z., Zhang Q. (2021). An Immune-Related Gene Prognostic Index for Triple-Negative Breast Cancer Integrates Multiple Aspects of Tumor-Immune Microenvironment. Cancers.

[35] Loi S., Drubay D., Adams S., Pruneri G., Francis P.A., Lacroix-Triki M., Joensuu H., Dieci M.V., Badve S., Demaria S. (2019). Tumor-Infiltrating Lymphocytes and Prognosis: A Pooled Individual Patient Analysis of Early-Stage Triple-Negative Breast Cancers. J. Clin. Oncol..

[36] Fan Y., He S. (2022). The Characteristics of Tumor Microenvironment in Triple Negative Breast Cancer. Cancer Manag. Res..

[37] Fujimoto Y., Inoue N., Morimoto K., Watanabe T., Hirota S., Imamura M., Matsushita Y., Katagiri T., Okamura H., Miyoshi Y. (2020). Significant Association between High Serum CCL5 Levels and Better Disease-Free Survival of Patients with Early Breast Cancer. Cancer Sci..

[38] Gerratana L., Basile D., Buono G., De Placido S., Giuliano M., Minichillo S., Coinu A., Martorana F., De Santo I., Del Mastro L. (2018). Androgen Receptor in Triple Negative Breast Cancer: A Potential Target for the Targetless Subtype. Cancer Treat. Rev..

[39] Hwang K.T., Kim Y.A., Kim J., Park J.H., Choi I.S., Hwang K.R., Chai Y.J., Park J.H. (2020). Influence of Androgen Receptor on the Prognosis of Breast Cancer. J. Clin. Med..

[40] Teschendorff A.E., Miremadi A., Pinder S.E., Ellis I.O., Caldas C. (2007). An Immune Response Gene Expression Module Identifies a Good Prognosis Subtype in Estrogen Receptor Negative Breast Cancer. Genome Biol..

[41] Rody A., Karn T., Liedtke C., Pusztai L., Ruckhaeberle E., Hanker L., Gaetje R., Solbach C., Ahr A., Metzler D. (2011). A Clinically Relevant Gene Signature in Triple Negative and Basal-like Breast Cancer. Breast Cancer Res..

[42] Criscitiello C., Bayar M.A., Curigliano G., Symmans F.W., Desmedt C., Bonnefoi H., Sinn B., Pruneri G., Vicier C., Pierga J.Y. (2018). A Gene Signature to Predict High Tumor-Infiltrating Lymphocytes after Neoadjuvant Chemotherapy and Outcome in Patients with Triple-Negative Breast Cancer. Ann. Oncol..

