# Supplementary material for: Prognostic Capability of TNBC 3-Gene Score among Triple-Negative Breast Cancer Subtypes
Source: Cancers (Basel). 2022 Sep 1;14(17):4286. doi: 10.3390/cancers14174286 (PMC9454544; doi:10.3390/cancers14174286)
Supplement: Supplementary file 1 [file cancers-14-04286-s001.zip › cancers-1841955-supplementary.pdf]

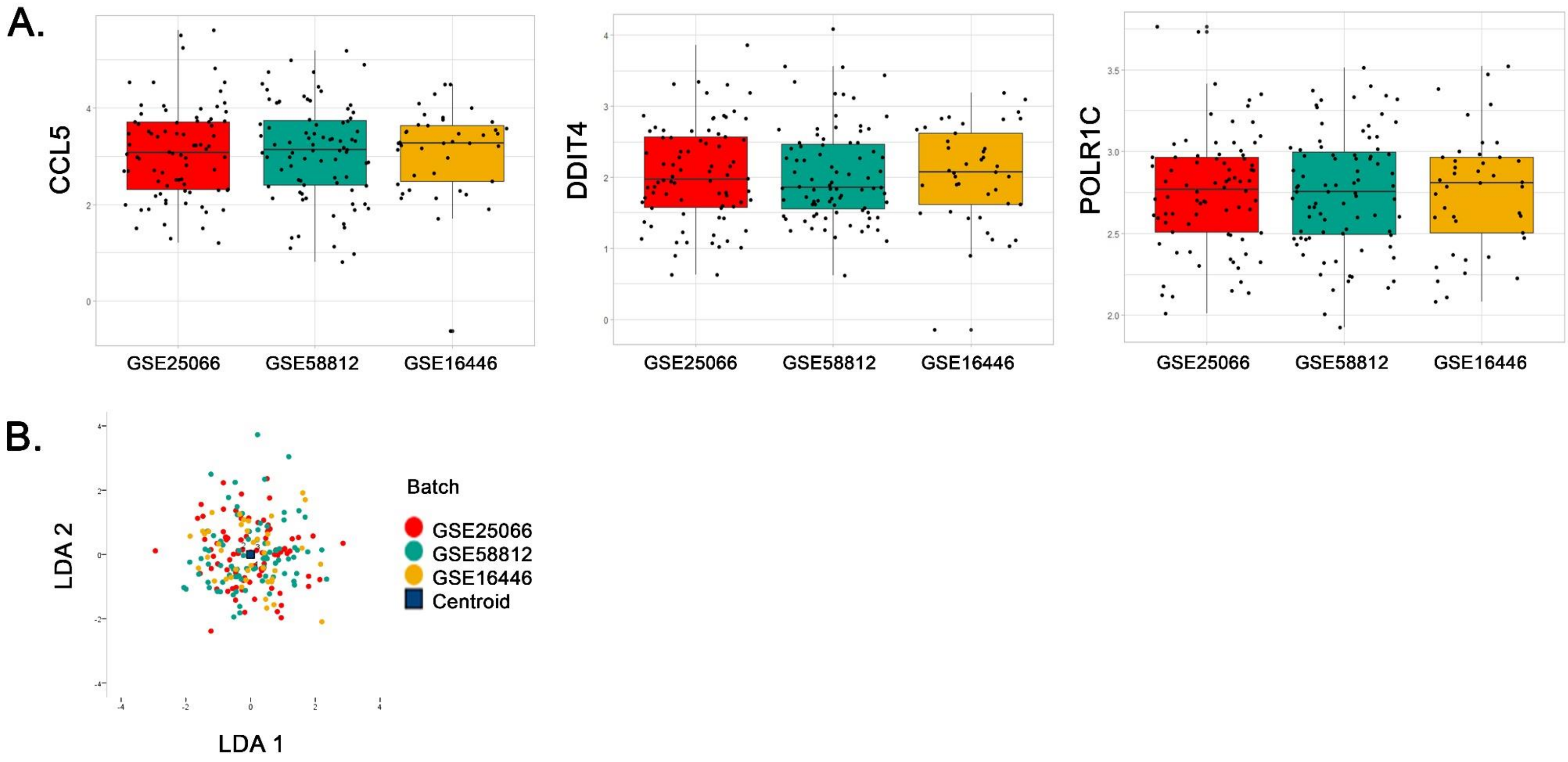

Figure S1.- Distribution of *CCL5, DDIT4* and *POLR1C* gene expression in three datasets, GSE25064 (n=80), GSE58812 (n=83) and GSE16446 (n=41). **A.** The expression of *CCL5* (p=0.869), *DDIT4* (p=0.830) and *POLR1C* (p=0.991) did not present significant differences between the three databases. **B.** The linear discriminant function (LDA) plot did not show a pattern of grouping in the data.
